# Investigation of a COVID-19 outbreak at a regional prison, Northern Uganda, September 2020

**DOI:** 10.11604/pamj.2022.43.10.33598

**Published:** 2022-09-06

**Authors:** Richard Migisha, Job Morukileng, Claire Biribawa, Daniel Kadobera, James Kisambu, Lilian Bulage, Alex Ndyabakira, Elizabeth Katana, Lisa A Mills, Alex Riolexus Ario, Julie R Harris

**Affiliations:** 1 Uganda Public Health Fellowship Program, Kampala, Uganda,; 2 Uganda Prisons Service, Kampala, Uganda,; 3 US Centers for Disease Control and Prevention, Kampala, Uganda,; 4 Ministry of Health, Kampala, Uganda,; 5 Division of Global Health Protection, Center for Global Health, US Centers for Disease Control and Prevention, Atlanta, USA

**Keywords:** COVID-19, SARS-CoV-2, prison, disease outbreaks, Uganda

## Abstract

Despite implementing measures to prevent introduction of COVID-19 in prisons, a COVID-19 outbreak occurred at Moroto Prison, northern Uganda in September 2020. We investigated factors associated with the introduction and spread of COVID-19 in the prison. A case was PCR-confirmed SARS-CoV-2 infection in a prisoner/staff at Moroto Prison during August-September 2020. We reviewed prison medical records to identify case-patients and interviewed prison and hospital staff to understand possible infection mechanisms for the index case-patient and opportunities for spread. In a retrospective cohort study, we interviewed all prisoners and available staff to identify risk factors. Data were analyzed using log-binomial regression. On September 1, 2020, a recently-hospitalized prisoner with unrecognized SARS-CoV-2 infection was admitted to Moroto Prison quarantine. He had become infected while sharing a hospital ward with a subsequently-diagnosed COVID-19 patient. A sample taken from the hospitalized prisoner on August 20 tested positive on September 3. Mass reactive testing at the prison on September 6, 14, and 15 revealed infection among 202/692 prisoners and 8/90 staff (overall attack rate=27%). One prison staff and one prisoner who cared for the sick prisoner while at the hospital re-entered the main prison without quarantining. Both tested positive on September 6. Food and cleaning service providers also regularly transited between quarantine and unrestricted prison areas. Using facemasks >50% of the time (adjusted risk ratio [aRR]=0.26; 95%CI: 0.13-0.54), or in combination with handwashing after touching surfaces (aRR=0.25; 95%CI: 0.14-0.46) were protective. Prisoners recently transferred from other facilities to Moroto Prison had an increased risk of infection (aRR=1.50; 95%CI: 1.02-2.22). COVID-19 was likely introduced into Moroto Prison quarantine by a prisoner with hospital-acquired infection and delayed test results, and/or by caretakers who were not quarantined after hospital exposures. The outbreak may have amplified via shared food/cleaning service providers who transited between quarantined and non-quarantined prisoners. Facemasks and handwashing were protective. Reduced test turnaround time for the hospitalized prisoner could have averted this outbreak. Testing incoming prisoners for SARS-CoV-2 before quarantine, providing unrestricted soap/water for handwashing, and universal facemask use in prisons could mitigate risk of future outbreaks.

## Introduction

Coronavirus Disease-19 (COVID-19), caused by severe acute respiratory syndrome coronavirus 2 (SARS-CoV-2), is an emerging challenge in prisons. Even when prisons ‘lock down’, multiple opportunities exist for introducing the virus into prisons, including visits from legal representatives and family members, transfer of prisoners between prisons, movement of prisoners to medical facilities outside the prison and to court appearances, and daily ingress and egress of prison staff [1]. In addition, crowded conditions, poor hygiene, and higher background prevalence of co-morbidities (e.g., tuberculosis, HIV) relative to other community settings may amplify both the risk of spread of SARS-CoV-2 virus and the risk of clinical complications among inmates in prisons [2,3]. To date, several large outbreaks have been reported among prisons in the USA [4-7], Brazil [8], Italy [9], India, South Korea, Pakistan, China, and the Philippines [10].

COVID-19 mitigation strategies in prisons have generally been similar to those employed in general populations, including measures to improve personal hygiene, physical distancing, suspension of social visits, health education, mass testing, and use of personal protective equipment (PPE) [1]. Some prisons have decarcerated prisoners jailed for minor offences or with short times remaining in their sentence as a way of reducing crowding [11]. However, the effectiveness of these measures in confined and overcrowded populations may be limited [12] Additional mitigation measures including routine testing, even for asymptomatic persons [6], isolation of individuals at high-risk for severe disease [13], improving ventilation systems [14], and embracing more participatory approaches in control measures where prisoners and other stakeholders are involved (such as health education campaigns in prisons using a peer-to-peer approach) may be required [11,15]

On March 21, 2020, Uganda confirmed its first case of COVID-19 in an incoming traveler from Dubai [16]. Effective April 2020, Uganda Prisons Service (UPS) began implementing multiple measures to reduce the risk of introduction and spread of COVID-19 into the prison system, including mandatory two-week quarantine for entering prisoners; limiting the number of visitors, including religious groups, relatives, and other community members; temperature screening for all persons entering prison, and providing handwashing facilities at entrances to prisons and prison wards (dormitories) [17]. On June 8, 2020, Uganda recorded its first prison case of COVID-19 in a prisoner in Namalu Prison, Nakapiripirit District. As of August 15, 2020, 159 COVID-19 cases had been reported in Ugandan prisons, with no deaths.

Following identification of an index case-patient with COVID-19 in Moroto Prison, Moroto District, on September 3, 2020, all prisoners and staff in the prison were tested on September 6, 2020 (testing of contacts of the quarantined index case) or September 14-15, 2020 (mass testing of all remaining prisoners and staff), identifying 202 SARS-CoV-2-infected prisoners and 8 infected staff. We investigated the outbreak to identify factors associated with the introduction and spread of infection in Moroto Prison and to recommend control and preventive measures for the future.

## Methods

**Study Setting:** Moroto Prison is the main prison in the Moroto region, in northern Uganda, and has a capacity of approximately 690 prisoners and 90 staff persons. The prison is located in Moroto District, about 528 kilometres by road northeast of Kampala, Uganda's capital and largest city. Starting in April 2020, the prison designated quarantine and isolation areas to accommodate incoming prisoners and confirmed COVID-19 cases.

**Case definition and identification:** we defined a confirmed case as a positive real-time RT-PCR test for SARS-CoV-2 in a prisoner or staff of Moroto Prison from August-September, 2020. Uganda Central Public Health Laboratory, located in Kampala, conducted laboratory testing of nasopharyngeal swabs using real-time RT-PCR (Berlin protocol) [18]. We reviewed Moroto Prison's medical records to identify case patients and non-cases at the prison. We also reviewed medical records at Moroto Regional Referral Hospital to obtain data on admitted prisoners/prison staff with confirmed SARS-CoV-2 infection at the hospital during August-September 2020.

**Environmental assessment:** we toured the prison premises to understand the prison setting and quarantine/isolation facilities. In addition, we interviewed the head of medical services at the prison to gather data on the initial cases of COVID-19 in the prison. We also interviewed police staff at Moroto Police Station to understand the flow of new prisoners from the community to the prison to identify additional opportunities for the introduction of COVID-19 into the prison system.

**Retrospective cohort:** we conducted a retrospective cohort study with all consenting prisoners, prison staff, and all on-site family members of prison staff; only family members aged =18 years were included. In Uganda, prison staff often reside in staff quarters on the prison campus with their family members, including spouses. We administered a standardized semi-structured questionnaire to collect socio-demographic data, including age, sex, occupation (prisoner, staff, or staff family member), and ward/dormitory of residence. Clinical data collected included date of sample collection for COVID-19 testing, COVID-19 test results, presence of symptoms at the time of sample collection, date of symptom onset, and presence of underlying medical conditions. Since there was a cholera outbreak around the same time at the prison, we also included a question about whether respondents had been diagnosed with cholera in the previous two months. We collected data on possible exposures to SARS-CoV-2, including a history of recent court appearances, handwashing practices, frequency of facemask use, history of recent transfer from another prison, and level of interaction with the local community. We also collected data on whether or not the prisoners belonged to a privileged prisoner group with special rights (“party”). Respondents were considered to belong to a party/privileged group if they had specific roles assigned to them inside or outside the prison, such as collecting firewood, fetching water, cooking, and herding cattle. With regard to the frequency with which respondents practiced facemask use and handwashing, ‘Always’ indicated more than 95% of the time; ‘Most of the time´ indicated >50-95%; ‘Occasionally’ indicated 20%-50%, and ‘Rarely´ indicated <20% [19].

Data were entered into Epi Data 3.1 (EpiData, Odense, Denmark) and exported to STATA 15 (StataCorp, College Station, Texas, USA) for analysis. We calculated attack rates using prison population data as of August 31, 2020, representing the prison population before many prisoners escaped on September 16, 2020. To calculate Risk Ratios (RRs) for factors associated with contracting COVID-19 at the prison, we performed univariable and multivariable analysis using log-binomial regression. All factors with p<0.2 in univariable analysis were included in the multivariable models. We examined “Personal Protective Measures” variables (handwashing and facemask use) singly and in combination to identify synergistic protective exposures. Age and sex were included in the final model even if not statistically significant in univariable analysis due to known interaction with the risk of SARS-CoV-2 infection and disease severity. Corresponding adjusted risk ratios (aRRs) and 95% confidence intervals were reported.

**Ethics approval and consent to participate:** COVID-19 in Uganda was declared a public health emergency and the Uganda Ministry of Health (MoH) gave the directive to investigate the COVID-19 outbreak in the prison upon request from Uganda Prisons Services (UPS). We obtained permission from UPS and Moroto Prison authorities to conduct this investigation. We sought verbal consent from all respondents before data collection. In an effort to minimize the risk of spreading COVID-19 infection, we did not obtain written informed consent, as the prison and MoH Standard Operating Procedures (SOPs) discouraged the exchange of materials by hand. Prospective participants were told that their participation was voluntary and that there would be no negative consequences if they refused to participate. During data collection, respondents were assigned unique identifiers instead of names to protect their confidentiality. Information was stored in password-protected computers and was not shared with anyone outside the investigation team. The Office of Science, U.S. Centers for Disease Control and Prevention, determined that this activity was conducted in response to a public health emergency and with the primary intent of public health practice (epidemic disease control activity). It was determined therefore to not be human subjects research.

**Availability of data and materials:** the datasets upon which our findings are based belong to the Uganda Public Health Fellowship Program and Uganda Prisons Service. For confidentiality reasons, the datasets are not publicly available. However, the data sets can be availed upon reasonable request from the corresponding author and with permission from the Uganda Public Health Fellowship Program, and Uganda Prisons Service.

## Results

### Index case and introduction of SARS-CoV-2 into the prison, September 2020

**Figure 1:**
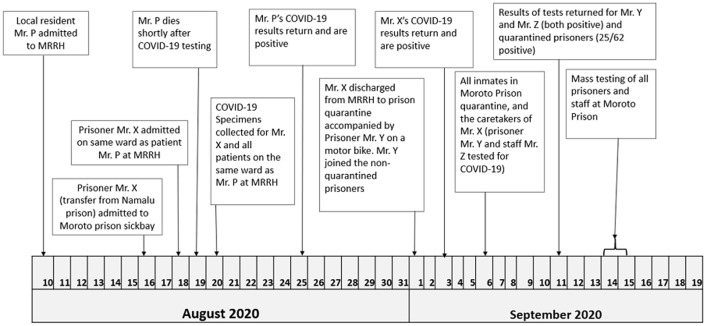
timeline of events in Moroto Prison and Moroto Regional Referral Hospital (MRRH) in Uganda during August-September 2020

The outbreak was initially linked to a male prisoner in his early thirties (Mr. X) (Figure 1). Mr. X was transferred from Namalu Prison in eastern Uganda to Moroto Prison for assessment and medical care for a respiratory illness that was unspecified at that time. Mr. X was later admitted to Moroto Regional Referral Hospital (MRRH) due to his poor clinical condition. During his admission, he was cared for by a fellow prisoner (Mr. Y) and guarded by a prison warden (Mr. Z). Mr. X was admitted to the same MRRH ward as another patient (Mr. P), who was suffering from a pneumonia-like illness. Mr. P and Mr. X shared a room from August 17-19. Mr. P died on August 19, very shortly after he was tested for COVID-19. All patients in the ward and their caretakers, except for Mr. X´s caretakers, had samples taken for COVID-19 testing on August 20, 2020. On August 25, 2020, a positive COVID-19 test result was returned for Mr. P. Mr. X, who had received a negative sputum test for tuberculosis, was treated for presumptive pneumonia.

Despite his pending COVID-19 test result and symptomatic status (coughing), Mr. X was discharged back to Moroto Prison on September 1, 2020. Upon discharge from MRRH, Mr. X and Mr. Y (his caretaker) boarded a motorcycle driven by Mr. Z (the prison warden guarding Mr. X in the hospital) to return to Moroto prison. As a new admission to the prison, Mr. X was placed in quarantine, as were all recent prisoner arrivals to Moroto Prison; there were 68 other prisoners in the same quarantine room at the time. Although the quarantine room was physically separate from the rest of the prison buildings, food was served to quarantined prisoners and water was brought to them in jerrycans by the same prisoners who provided food and water to the non-quarantined prisoners.

Mr. X received a positive COVID-19 test result on September 3, 2020 (15 days after being tested), while he was in quarantine at Moroto Prison. He was taken to Jinja Regional Referral Hospital (JRRH) for isolation and treatment. During this time, he was diagnosed with comorbid M. tuberculosis infection based on a sample taken from the pleural aspirate. This was considered the likely agent of his original illness, despite his previous negative sputum test. To rule out suspicion of COVID-19 introduction from the original Namalu Prison, all inmates at Namalu Prison and the two prison staff persons who transported Mr. X from Namalu Prison to Moroto Prison underwent PCR testing for COVID-19 on 12 September 2020. All tested negative. The caretaker (Mr. Y) and the prison warden who guarded Mr. X at the hospital (Mr. Z) tested positive for COVID-19 from swabs taken on September 6, 2020 (12 days after Mr. P´s COVID-19 test came back positive). Neither Mr. Y nor Mr. Z was quarantined at the prison upon return from the MRRH hospital and both interacted immediately with the rest of the prison population.

### Descriptive epidemiology of COVID-19 case-patients, and testing at Moroto Prison, Uganda, September 2020

On September 6, 14, and 15, 2020, all 782 prisoners and staff/family members at Moroto Prison underwent testing for SARS-CoV-2 infection. Among 68 persons tested on September 6, 25 (37%) were positive, while among 714 persons tested on September 14-15, 185 (26%) were positive). In total, 210 (202 prisoners and 8 staff) tested positive, for an overall attack rate of 27%. Attack rates ranged from 7% among all prison staff to 41% among quarantined prisoners (Table 1).

**Table 1:** attack rates among tested prisoners and staff by sex, age, and occupation during the outbreak of COVID-19 in Moroto Prison, Uganda, September 2020

Population category	Population*	Number of cases	Attack Rate/100 persons
All prisoners	692	202	29
Male prisoners	677	197	29
Female prisoners	15	5	33
Privileged group˫	69	21	30
Prisoners in quarantine	63	26	41
All staff	90	8	9
Male staff	71	8	11
Female staff	19	0	0
All prisoners and staff	782	210	27

˫prisoners with special tasks/privileges, such as cooking, fetching firewood, cleaning, and herding cattle; *The denominator is the tested population

**Figure 2:**
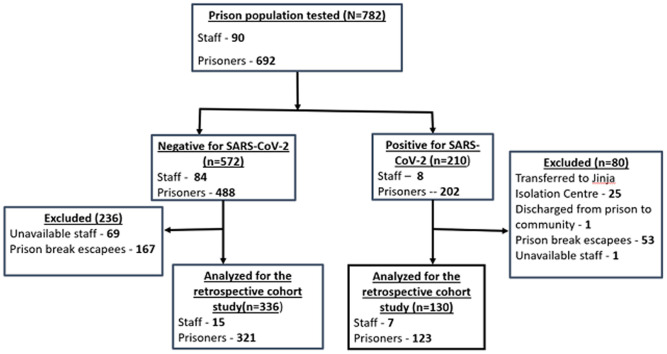
flow diagram for the inclusion and exclusion of prisoners and staff of Moroto Prison into the retrospective cohort study analysis, Moroto Prison COVID-19 outbreak, September 2020

**Figure 3:**
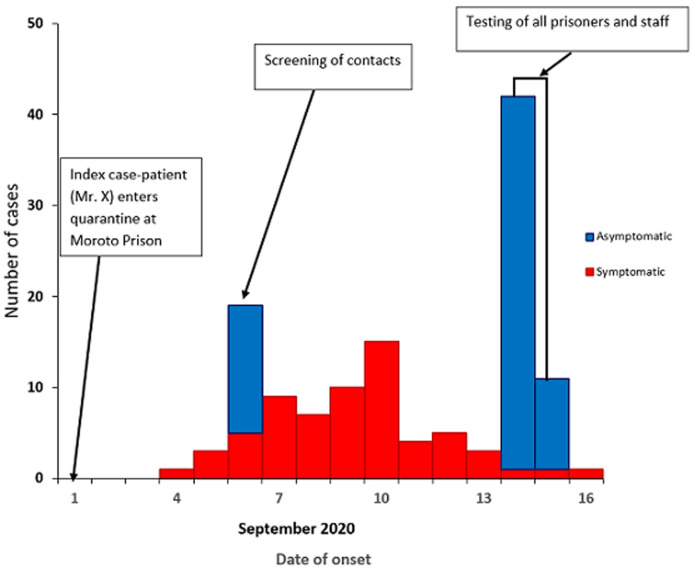
SARS-CoV-2 infection among prisoners and staff, Moroto Prison, Uganda, by date of symptom onset during September 2020 (n=130)

On September 16, 220 prisoners (including 53 who had tested positive) escaped the prison, citing fears of ‘dying in prison with COVID-19’. As a result, 562 persons remained for interviews, of whom we interviewed 466 (444 prisoners and 22 staff) (Figure 2). Reported illness onset for symptomatic case-patients at Moroto Prison ranged from September 4-16, 2020 (Figure 3). The median age of the respondents was 30 (range, 18-67) years (Table 2).

**Table 2:** sociodemographic, clinical, and behavioural characteristics of interview respondents, Moroto Prison, Uganda, September 2020

Variable	Total cohort (N=466)	
	Frequency	Percent
**Age category in years**		
18-29	226	48
30-49	184	39
≥50	56	12
Male sex	446	96
**Status**		
Prisoner	444	95
Staff/relative	22	5
**Duration in Moroto prison (n=438)**		
≤2 months	115	26
>2 months	323	74
Had sample taken	466	100
Tested positive	130	28
Had symptoms at sample collection§	61	47
**Underlying medical conditions**		
HIV	15	3.2
Hypertension	6	1.3
Neurological disease	5	1.1
Liver disease	2	0.4
Pregnancy	1	0.2
Lung disease	1	0.2
Asthma	1	0.2
Heart disease	1	0.2
At least one underlying medical condition	32	6.7
Belongs to a privileged˫ group (‘work party’)*	69	16
Transferred to Moroto from another prison*	244	52
Went to court in past 2 months	53	11
Ever treated for TB	28	6.0
Current smoker	62	13
Diagnosed with cholera during Moroto outbreak	27	5.8
Use of personal protective measures		
None˨	21	5
Facemask use sometimes/occasionally	90	19
Facemask use always/most of the time	88	19
Handwashingδ	9	2
Handwashingδ and ever used facemask	258	55

§Denominator is 130 case-patients; *Denominator is Prisoners only (n=444);

˫prisoners with special tasks/privileges such as cooking, fetching firewood, cleaning, and herding cattle

˨ Did not wash hands with soap and water after touching surfaces and never used facemasks

δ Handwashing with soap and water after touching surfaces

### Clinical features of case-patients at Moroto Prison, Uganda, September 2020

Of the 130 patients who tested positive and who remained in prison, 69 (53%) reported no symptoms. The most common symptoms reported were headache (27%), cough (25%), subjective fever (24%), runny nose (17%), and chest pain (16%) (Table 3). None of the case patients died.

**Table 3:** clinical features among 130 COVID-19 case-patients at Moroto Prison, Uganda, September, 2020

	Total (N=130)	
Symptom	Frequency	Percent
Asymptomatic	69	53
Headache	35	27
Cough	32	25
Fever	31	24
Runny nose	23	18
Chest pain	21	16
Muscle pains	16	12
Sore throat	14	11
Joint pains	7	5
Shortness of breath	3	2
Nausea	3	2
Anosmia	1	1

### Environmental assessment and key informant interviews

#### Prisoner entry into Moroto Prison, Uganda, September 2020

Following a person´s arrest in Uganda, they usually spend a short time (guidelines stipulate no more than 48 hours, but in practice, this time may be longer) in a cell at the police station before being charged in court and admitted to prison. Police cells may house up to 20 prisoners in a small space. Since April 2020, incoming prisoners to Moroto Prison were subjected to quarantine for a minimum of 14 days, after which they mixed with other prisoners if they did not develop any COVID-19-like symptoms. COVID-19 testing during quarantine was not done unless the individual under quarantine reported symptoms. In addition, all prisoners and staff received a facemask for use at the prison. However, while face mask used in the prison was encouraged, it was not enforced.

### Moroto Prison organization, September 2020

**Figure 4:**
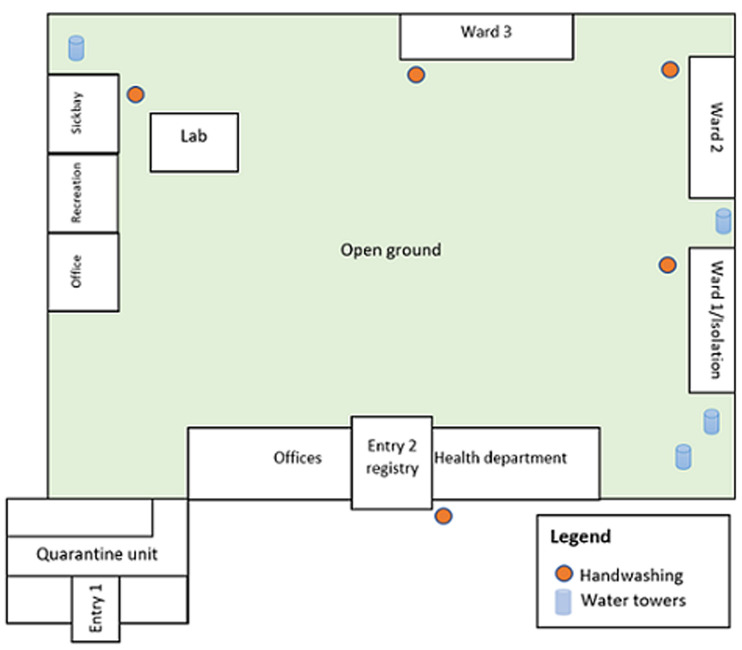
diagram of the setup of Moroto Prison, Uganda, as of September 2020; ward refers to a prison unit that acts as a dormitory

The prison has three wards, a sick bay, a quarantine centre, and an isolation centre (Figure 4). There were handwashing points at all entrances to the prison, wards, and the sick bay. The wards are large open dormitory-style spaces in which prisoners sleep on floor mats near each other. Ward 1 was designated as the isolation centre during the outbreak. Each male ward is approximately 20m x 8m and accommodates ~200 inmates; the sickbay accommodates 20; the quarantine center 60 (ideal capacity was 40); and the female ward 15. The quarantine centre at Moroto Prison (size: 7m x 9m) included two admission rooms. Each room received new admissions until it reached full capacity before the 14 days of quarantine could begin for all prisoners. The quarantine area was poorly ventilated with small vents near the ceiling. The duration of stay for most prisoners in quarantine typically ranged from 4-8 weeks. There was a cholera outbreak at the quarantine center from August 22-September 3, 2020.

Although the prison was still under COVID-19-related lockdown and additional security due to the recent escape, prisoners who belonged to prison parties´ and prison staff members had opportunities to interact with the local community and return to the prison throughout the entire period.

### Hypothesis generation findings

Based on the descriptive epidemiology and the interviews, we suspected that the primary case and/or the caretaker and guard were the likely sources of the introduction of COVID-19 into the prison. We hypothesized that the outbreak could have been amplified by service staff shared between the quarantined and regular prisoners. Failure to use protective measures (masks, handwashing) was also considered a contributor to transmission.

### Risk factors for contracting COVID-19 in Moroto Prison, Uganda, September 2020

In multivariable analysis (Table 4), prisoners who were recently transferred to prison had a 50% increased risk of contracting COVID-19 (aRR=1.50). Self-reported ever-use of facemasks along with performing handwashing after touching surfaces (aRR=0.25) was protective against contracting COVID-19. Self-reported use of facemask always/most of the time was protective (aRR=0.26).

**Table 4:** risk factors for contracting COVID-19 among prisoners and staff, Moroto Prison, Uganda, September 2020

	COVID-19 status (n, %)		Unadjusted	Adjusted
Variable	Positive (N=130)	Negative (N=336)	cRR (95%CI)	aRR (95%CI)
**Age category in years**				
18-29	55 (42)	171 (51)	Ref	Ref
30-49	61 (46)	123 (37)	1.36 (1.0-1.9)	1.24 (0.84-1.84)
≥50	14 (10)	42 (13)	1.03 (0.62-1.71)	1.12 (0.61-2.06)
**Sex**				
Male	126 (97)	320 (95)	Ref	Ref
Female	4 (3)	16 (5)	0.71 (0.29-1.72)	0.87 (0.32-2.40)
Belongs to a privileged group/party*				
No	102 (83)	273 (85)	Ref	
Yes	21 (17)	48 (15)	1.12 (0.76-1.66)	
Transferred to Moroto prison in past 2 months*				
No	40 (33)	160 (50)	Ref	Ref
Yes	83 (68)	161 (50)	1.70 (1.22-2.36)	1.50 (1.02-2.22)
Went to court in past 2 months*				
No	112 (91)	279 (87)	Ref	
Yes	11 (9)	42 (13)	0.73 (0.42-1.28)	
**Ever treated for TB**				
No	121 (93)	317 (94)	Ref	
Yes	9 (7)	19 (6)	1.16 (0.67-2.04)	
**Current smoker**				
No	116 (89)	288 (86)	Ref	
Yes	14 (11)	48 (14)	0.79 (0.48-1.28)	
**Use of personal protective measures**				
None**˨**	15 (12)	6 (2)	Ref	Ref
Facemask use sometimes/occasionally	48 (37)	42 (13)	0.75 (0.54-1.05)	0.70 (0.38-1.27)
Facemask use always/most of the time	16 (12)	72 (21)	0.25 (0.15-0.43)	0.26 (0.13-0.54)
Handwashing**δ**	6 (5)	3 (1)	0.93 (0.55-1.59)	0.96 (0.37-2.48)
Handwashing**δ** and ever used facemask	45 (35)	213 (63)	0.24 (0.17-0.36)	0.25 (0.14-0.46)
**Diagnosed with cholera in last 2 months**				
No	125 (96)	314 (93)	Ref	
Yes	5 (4)	22 (7)	0.65 (0.29-1.45)	
**HIV status**				
Negative	123 (95)	327 (97)	Ref	
Positive	7 (5)	9 (3)	1.60 (0.90-2.85)	

cRR: Crude Risk Ratio; aRR: Adjusted Risk Ratio; CI: Confidence interval; Ref: Reference category; TB: Tuberculosis

*Assessed only among 444 prisoners (123 positive and 231 negative for COVID-19)

˨ Did not wash hands with soap and water after touching surfaces and never used facemasks

**δ** Handwashing with soap and water after touching surfaces

## Discussion

The COVID-19 outbreak at Moroto Prison during September 2020 was likely introduced in the prison by a prisoner who was discharged from a nearby hospital, where he likely contracted COVID-19 from a sick patient before entering quarantine at the prison, and/or by his prison caretakers who stayed with him at the hospital. The prisoner had shared a room with a COVID-19 patient, who later died. More than 4 in 10 quarantine prisoners and nearly 3 in 10 non-quarantined prisoners were infected. All cases (beyond the index case) were identified through contact testing and mass testing, with approximately half being asymptomatic. However, individuals who used a facemask >50% of the time, or who reported any facemask use in combination with handwashing, were significantly less likely to contract COVID-19 than persons who did neither. Two persons who cared for the index case-patient in the hospital returned to the prison without being quarantined and later tested positive for SARS-CoV-2 infection. These two individuals might have concurrently introduced the virus into the non-quarantine wards of the prison. However, the high attack rate among prisoners in quarantine suggests that this may have been the first point of entry for the virus into the prison. The delayed turnaround time of test results for the primary case patient and failure to test his caretakers facilitated the spread of the virus.

Following the entry of the index case patient to quarantine, a large number of cases became symptomatic and/or tested positive within one incubation period. The concurrence of tuberculosis and COVID-19 in the index patient may have increased the transmission risk. However, the crowded living conditions in quarantine also likely facilitated the spread. The quarantine space was small and overcrowded; although it had small vents in the upper area near the ceiling, it was not well-ventilated. This is a common issue with prisons in general [2]. Other airborne diseases, such as tuberculosis, also spread effectively in prisons [3]. Improving ventilation systems in prisons could reduce the risk of the spread of such respiratory diseases, including COVID-19.

SARS-CoV-2 infection may have spread to other areas of the prison in one of two ways. First, there may have been simultaneous introductory events in the general prison and in the quarantine. Second, the prisoner and staff member who cared for and guarded the index case during his hospitalization at MRRH (Mr. Y and Mr. Z) were not subjected to quarantine on their return and mixed freely with the rest of the prison population. Both developed COVID-19, which could have occurred due to hospital exposures during the caretaking of Mr. X or after their return to the prison from other subsequently-infected prisoners. Provision of personal protective equipment (PPE), such as facemasks for caretakers who need to enter and exit the prison, may reduce the risk of introducing infection from staff, and may ensure that persons who interact with the community outside the prison undergo quarantine before mixing with the rest of the prison population. It is also possible that the infection spread from quarantine to the general prison population via the prisoners who provided food and water to both the quarantined prisoners and the rest of the prison population. Measures such as separating prisoners/staff who provide services to quarantined prisoners from those who provide services to the general prison population and enforcing the use of personal protective equipment by the service providers could also reduce the risk of spread.

Prior to this outbreak investigation, prisoners in quarantine were not tested for SARS-CoV-2 infection before exiting quarantine unless they developed symptoms. However, half of the infected prisoners in this study were asymptomatic. This symptom-based release from quarantine provides opportunities for asymptomatic, infected persons to introduce COVID-19 into the general prison population. Quarantining cohorts in smaller numbers could reduce the number of days spent by an individual in quarantine (range was 4-8 weeks for prisoners in this study due to the constant influx of new prisoners into quarantine, requiring restarting of the 2-week period), and testing prisoners before release from quarantine might help limit the scope of outbreaks and prevent future outbreaks. Longer durations of quarantine increase the risk of large numbers of infected persons, as new inmates enter the quarantine and mix with persons who would otherwise have been unexposed [1]. Furthermore, testing prisoners for SARS-CoV-2 before they are admitted to quarantine could further reduce the chances that the virus is being introduced into the quarantine, as occurred in this outbreak.

Lengthy turnaround time for COVID-19 test results for the prisoner index case-patient may have been the most important factor in this outbreak. The results of his COVID-19 test were not returned until 14 days after it was conducted, after which he had already returned to prison. Introducing exposed persons with pending COVID-19 tests to prisons - even to quarantine - should be avoided. If the return is necessary, ensuring solitary quarantine until test results are available is clearly preferable to mixing a potentially infected person with non-infected people. In addition, continued efforts to reduce turnaround times for SARS-CoV-2 tests could help avert similar situations in the future. This may necessitate decentralization of laboratory testing for SARS-CoV-2 and dedicating regional laboratory hubs for detention facilities to carry out the tests for SARS-CoV-2 in the country. Point-of-care testing (such as through GenXpert, already available in some prison health facilities) could be explored. Despite the pitfalls of rapid diagnostic tests (lower sensitivity compared with PCR and a short window of infection during which they are useful) [20], they may assist in more quickly identifying and isolating symptomatic cases. It is challenging to address all possible entry routes for COVID-19 into institutions.

Importantly, we found that adherence to ‘low-tech’ personal preventive measures such as facemask use and hand washing after touching surfaces were significantly protective, reducing infection risk by as much as 70-80%. This finding is in agreement with several studies elsewhere that have shown hand hygiene and mask used to be associated with a reduced risk of contracting COVID-19 in general populations [21-23]. Some detention facilities may restrict access to alcohol-based sanitizers, disinfectants, and soap during ‘normal times’ [1]. Therefore, during the COVID-19 pandemic, improved access to face masks and maximizing their use, and free and easy access to water and soap is warranted in detention facilities to improve infection prevention and control. Consideration of practical approaches to facilitate social distancing, such as additional outdoor time, could also be useful.

**Limitations:** the findings from this investigation have some limitations. First, we conducted this investigation at a time when a prison escape event had recently taken place; 24% of non-cases and 25% of cases had escaped. Thus, we were unable to assess exposures in all prisoners. However, this is unlikely to have biased our identified associations since cases and non-cases escaped in equal proportions. Second, self-reporting of exposures and symptoms is susceptible to social desirability bias since prisoners may fear discrimination, punishment, and being medically isolated [24] if they report exposures or symptoms. This may have led to under-ascertainment of exposures and symptoms among the respondents in this investigation. Nonetheless, our analyses provided useful information to the Uganda Prison Service on events associated with COVID-19 spread in prison settings.

**Funding and disclaimer:** this project was supported by the President´s Emergency Plan for AIDS Relief (PEPFAR) through the US Centers for Disease Control and Prevention Cooperative Agreement number GH001353-01 through Makerere University School of Public Health to the Uganda Public Health Fellowship Program, MoH. The contents of this manuscript are solely the responsibility of the authors and do not necessarily represent the official views of the US Centers for Disease Control and Prevention, the Department of Health and Human Services, Makerere University School of Public Health, or the MoH. The staff of the funding body provided technical guidance in the design of the study, ethical clearance and collection, analysis, and interpretation of data and in writing the manuscript, but did not have any contact with participants or access to data with identifiers. The use of trade names and commercial products is for identification only and does not imply endorsement by the US Centers for Disease Control and Prevention.

## Conclusion

SARS-CoV-2 was likely introduced into the prison quarantine unit by a prisoner arriving after acquiring infection in the regional referral hospital. COVID-19 likely spread via shared food servers or infected prison staff and prisoners who cared for the index prisoner case at the hospital and returned to the prison without quarantining. Subsequent sub-optimal use of personal protective measures among prisoners and staff likely promoted infection spread. Mitigating measures, such as COVID-19 testing with a review of results before admission of prisoners to group quarantine units (and individual isolation until results have returned negative), unrestricted access to soap and water for handwashing, and ongoing provision of sufficient facemasks in prisons may avert or reduce the impact of future similar outbreaks.
